# Distinct functions of HTLV-1 Tax1 from HTLV-2 Tax2 contribute key roles to viral pathogenesis

**DOI:** 10.1186/1742-4690-6-117

**Published:** 2009-12-17

**Authors:** Masaya Higuchi, Masahiro Fujii

**Affiliations:** 1 Division of Virology, Niigata University Graduate School of Medical and Dental Sciences, 1-757 Asahimachi-Dori, Niigata 951-8510, Japan

## Abstract

While the human T-cell leukemia virus type 1 (HTLV-1) is the etiologic agent of adult T-cell leukemia/lymphoma (ATL), to date, its close relative HTLV-2 is not associated with ATL or other types of malignancies. Accumulating evidence shows that HTLV-1 Tax1 and HTLV-2 Tax2 have many shared activities, but the two proteins have a limited number of significantly distinct activities, and these distinctions appear to play key roles in HTLV-1 specific pathogenesis. In this review, we summarize the functions of Tax1 associated with cell survival, cell proliferation, persistent infection as well as pathogenesis. We emphasize special attention to distinctions between Tax1 and Tax2.

## Background

Adult T-cell leukemia/lymphoma (ATL) is an aggressive form of leukemia/lymphoma characterized by the malignant proliferation of CD4 T cells infected with human T-cell leukemia virus type 1 (HTLV-1) [1-4]. HTLV-1 infection also causes a neurodegenerative disease termed HTLV-1-associated myelopathy/tropical spastic paraparesis (HAM/TSP) [5,6]. HTLV-1 belongs to the delta-retrovirus family, and it infects currently 10-20 million people in the world, especially in southwestern Japan, Africa, the Caribbean Islands and South America [7]. HTLV-1 transmission mainly occurs from mother to child through breast milk [8]. After the transmission and infection, HTLV-1 immortalizes the infected CD4 T-cells; and this immortalization establishes a life-long persistent infection in a host [9,10]. The immortalization of infected T-cells is likely to be dependent on cytokines [11] including interleukin (IL)-2, and perhaps also occurs in cytokine-independent (or less-dependent) ways as discussed later. Indeed, HTLV-1 transforms primary human CD4 T-cells in an IL-2-dependent as well as IL-2-independent manner *in vitro*. This transformation event of infected T-cells alone is, however, not sufficient for ATL development, since only a fraction of HTLV-1 infected individuals (approximately 5%) suffer ATL after a long latency period (60 years on average). Thus, both multiple genetic and epigenetic changes [12] in infected T-cells and the deterioration of host immune system are thought to be prerequisites for ATL development.

Intriguingly, a closely related delta-retrovirus, human T-cell leukemia virus type 2 (HTLV-2), does not cause any leukemia or lymphoma in infected people in spite of its ability to immortalize *in vitro *human T-cells in an IL-2-dependent manner as effectively as HTLV-1 [13]. Moreover, the association of HTLV-2 infection with HAM/TSP is quite rare. Thus, HTLV-2 should be regarded as defective in promoting certain steps of leukemogenesis and neurologic disease development, and this virus may be a useful comparative tool for understanding the pathogenic activities of HTLV-1. In addition, HTLV-3 and HTLV-4 have recently been identified from bushman hunters in central Africa, although the association of these viruses to human diseases needs further investigations [14-16].

**Figure 1:**
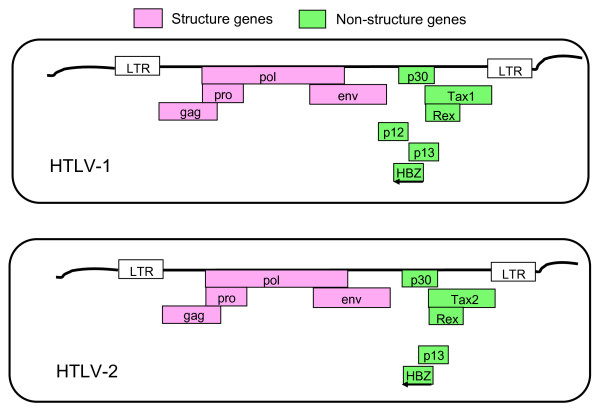
**Structures of HTLV-1 and HTLV-2**. A comparison of HTLV-1 genes with those of HTLV-2. Some HTLV-2 non-structural genes such as APH-2, the equivalent protein to HTLV-1 HBZ, are indicated as HBZ and are named using HTLV-1 nomenclature in this figure.

In addition to structure genes, *gag*, *pol*, and *env*, HTLV-1 encodes several non-structural genes including p12, p13, p30, Rex, and Tax (Figure 1) [17]. Among them, HTLV-1 Tax (Tax1) plays a central role in the immortalization of infected T-cells and the persistence of infection in a host. Tax1 immortalizes primary human T-cells in an IL-2 dependent manner, and transforms a T cell line CTLL-2 from IL-2 dependent growth into IL-2-independent growth [18-20]. In addition, Tax1 induces anchorage independent growth of a Rat-1 fibroblast cell line, and such cells can develop tumors in nude mice [21]. These results suggest that Tax1 has both immortalizing and oncogenic potentials.

Tax1 was originally identified as a transcriptional activator that interacts with a triplicated Tax-responsive enhancer in the 5' long terminal repeat (LTR) of the HTLV-1 genome [22,23]. In addition, through interacting with a number of cellular proteins, Tax1 functions in the survival of HTLV-1-infected T-cells, in cell cycle progression, cell growth, and in induced genomic instability [23-25]. All these Tax1 functions are thought to work cooperatively to transform HTLV-1 infected T-cells and have pivotal roles in the development of HTLV-1 associated diseases. Among them, Tax1-induced genomic instability is undoubtedly involved in ATL development, but we will not discuss this topic because it has been reviewed well elsewhere [26-28]. Here, we will focus on the functions of Tax1 in cell survival, proliferation, and pathogenesis, with an emphasis on distinctions between HTLV-1 and HTLV-2 Tax proteins.

### Activation of the NF-κB pathway

The NF-κB family of transcription factors, including RelA, c-Rel, RelB, NF-κB1 (p50 and its precursor p105), NF-κB2 (p52 and its precursor p100), plays a central role in immune functions, such as innate and adaptive responses to pathogens, survival of lymphocytes, and lymphoid tissue development [29]. On the other hand, aberrant NF-κB activation is implicated in the genesis of many types of cancer, especially hematologic malignancies such as leukemia, lymphoma, and myeloma [30]. There are two NF-κB-signaling pathways that regulate overlapping, but distinct sets of cellular genes; and these two pathways are called the canonical and the noncanonical NF-κB pathways. Inflammatory cytokines, genotoxic stress, antigens, and toll like receptor (TLR) stimulation activate the canonical pathway, whereas a subset of TNF family members, such as CD40L, lymphotoxin-β, BAFF, RANKL, and TWEAK activate the noncanonical pathway. While the activation of the canonical pathway results in the degradation of its inhibitor IκB and the translocation of the p50/RelA complex into the nucleus, the activation of the noncanonical pathway results in the processing of p100/RelB into p52/RelB and the translocation of the latter into the nucleus.

NF-κB activity is tightly controlled in normal T cells, and it is transiently activated in certain circumstances such as during immune stimulation. By contrast, NF-κB is constitutively active in HTLV-1-infected T cells [31-34]. This constitutive NF-κB activation is mediated by Tax, and the activity is essential for T cell transformation by HTLV-1 and HTLV-2. For instance, HTLV-1 and HTLV-2 carrying mutant *tax1 *and *tax2 *genes defective for NF-κB activation, can not immortalize primary human T cells [35,36] (however, in some cases of Tax over expression, NF-κB activity is not needed to immortalize primary human T cells [37]). In addition, Tax1 NF-κB mutants cannot transform CTLL-2 and Rat-1 cells, consistent with the importance of NF-κB activation for Tax1-induced cell growth promotion [20,38,39].

### Mechanism of NF-κB activation by Tax

Both Tax1 and Tax2 activate the canonical NF-κB pathway through interacting with multiple NF-κB regulators. While the full scheme of canonical NF-κB activation by Tax has not been elucidated yet, the activation of the IKK complex by Tax through binding with its scaffold subunit IKKγ (NEMO) is a central event. The above conclusion was established based on the observation that the loss of NEMO completely abrogates the activation of NF-κB by Tax1 [40,41]. A MAP3K, TAK1, stimulates IKK activity upon various stimuli such as TLR, IL-1, and anti-CD3 stimulation [42]. Tax1, through interacting with TAK1, increases TAK1 kinase activity [43]. Thus, Tax1 functions as an adaptor, mediating the TAK1-IKK interaction through binding to both molecules.

Tax1 undergoes several posttranslational modifications, including phosphorylation, acetylation, sumoylation, and ubiquitination [44-50]. Among these, Tax1 ubiquitination is crucial for its binding to NEMO and for the subsequent NF-κB activation [48]. Tax1 polyubiquitination is predominantly composed of K63-linked chains, and such ubiquitination is dependent on the E2 ubiquitin conjugating enzyme, Ubc13 [48]. In addition, another Tax1 binding protein TAX1BP1 forms a ternary complex with the E3 ubiquitin ligase Itch and the ubiquitin-editing enzyme A20. The TAX1BP1-A20 deubiquitinase complex is a negative regulator of NF-κB activity induced by inflammatory cytokines; Tax1 can disrupt this inhibitory complex to thereby trigger constitutive NF-κB activation in HTLV-1-infected cells [51,52]. Recently, it was found that NEMO-Related Protein (NRP/Optineurin) binds to both Tax1 and TAX1BP1; this newly-described interaction can positively modulate Tax1 ubiquitination and NF-κB activation [53].

### Tax1 specific activation of NF-κB2

**Figure 2:**
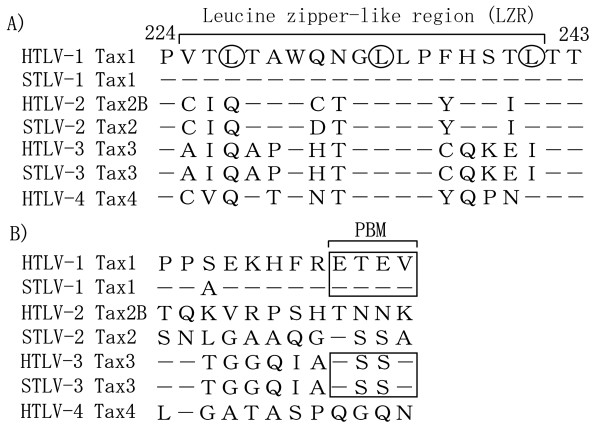
**Two regions of Tax1 are responsible for increased transforming activity relative to Tax2 in CTLL-2 cells**. (A) The amino acid sequences of the Tax LZR of HTLVs and STLVs. A bar indicates the identical amino acids of Tax from the other six viruses to that of Tax1. The leucine residues constituting a putative LZ structure are marked by circle. (B) The amino acid sequences in the C-terminal ends of the respective Tax proteins. The PBMs are surrounded by squares.

In addition to the canonical NF-κB pathway, Tax1 activates the noncanonical NF-κB pathway [54]. Tax1 simultaneously binds to the IKK complex and NF-κB2/p100, leading to IKKα mediated p100 phosphorylation and subsequent p100 processing into p52 [54]. Interestingly, HTLV-2 Tax2 cannot induce p100 processing into p52 when transiently expressed in the Jurkat T-cell line, although Tax2 can activate the canonical NF-κB pathway to a level comparable to Tax1 [55]. The major defect of Tax2 in p100 processing is an inability of Tax2 to interact with p100 [55]. The Tax1 region encompassing amino acids 225-232, overlapping with the leucine zipper like region (LZR), is responsible for Tax1-mediated p100 processing and p52 nuclear translocation (Figure 2) [56]. Since Tax1 LZR is not required for interaction with p100, Tax1 interaction with IKK complex and p100 is not sufficient for p100 processing. Thus, an as-yet-unidentified molecule which associates with the Tax1 LZR might be involved in the activation of the noncanonical NF-κB pathway.

The transforming activity of Tax1 towards CTLL-2 is much higher than that of Tax2, and such increased activity is partly mediated through Tax1-specific activation of the noncanonical NF-κB pathway [55-58]. For instance, the exogenous expression of an activator of the noncanonical pathway, a constitutively active NF-κB inducing kinase (NIK), restores the transforming activity of Tax2 to a level equivalent to that of Tax1 [55]. Interestingly, the requirement of NF-κB2 activation in Tax1 transformation appears to be T-cell specific, since the NF-κB2 activation by Tax1 is not needed in the transformation of Rat-1 [59]. Given the fact that aberrant activation of the noncanonical NF-κB pathway is associated with the development of mature T-cell leukemia and lymphoma [60], these results suggest that the activation of the noncanonical NF-κB pathway by Tax1 plays a role in HTLV-1 specific pathogenesis.

### NF-κB activation in T-cell transformation

Continued cell cycle progression and resistance to apoptosis are two fundamental functions associated with the transformation of HTLV-1 infected T cells. NF-κB activation by Tax1 is critically involved in both functions. When expressed in G1- arrested primary human T cells, Tax1 induces cell cycle progression from the G1 to S phase through activation of E2F transcription factors [61]. The ability of Tax1 to promote cell-cycle progression is at least partially mediated through the induction of cyclin D2 and Cdk6 via the NF-κB pathway [62]. The activation of both the canonical and noncanonical NF-κB pathways by Tax1 is involved in this process, since knockdown of either RelA or NF-κB2/p100 by short hairpin RNA reduces Tax1-induced cell-cycle progression [62]. It is noteworthy that in addition to this NF-κB dependent function, Tax1 activates Cdk4/6 through direct interaction with Cdk4, Cdk6, and Cdk inhibitors such as p16INK4A and p15INK4B [63-69]. Thus, the cell cycle progression induced by Tax1 requires both the induction of cell cycle regulators in an NF-κB-dependent manner and their subsequent activations in an NF-κB-independent manner.

Tax1 has been shown to confer resistance to apoptosis through inducing anti-apoptotic proteins such as Bcl-xL, survivin, cFLIP, xIAP, cIAP1, and cIAP2 in a NF-κB dependent manner [70-75]. Both the noncanonical and canonical NF-κB pathways appear to play positive roles in the inhibition of apoptosis. For instance, cell lines established from large granular leukemia developed in Tax1 transgenic mice are resistant to apoptosis inducers, whereas knockdown of either NF-κB1 or NF-κB2 augments apoptosis, through reducing the expression of the xIAP, cIAP1, cIAP2, and cFLIP [74].

### Inductions of cytokines, chemokines and receptors

Tax1 upregulates the expression of genes encoding cytokines, chemokines, cell surface ligands, and their receptors, in an NF-κB, AP-1, CREB/ATF and/or NFAT dependent manner. They include IL-2 receptor (IL-2R) α-chain, IL-9, IL-13, IL-15/IL-15R, IL-21/IL-21R, IL-8, CCL2, CCL5, CCL22, CCR9, CXCR7, CD40, OX40/OX40L, and 4-1BB/4-1BBL [76-94]. Among these, the IL-2R α-chain is crucially important for T-cell immortalization by Tax, since the immortalized cells are dependent on IL-2 for their growth.

Transient transfection studies showed that Tax1 induces the expression of IL-2 through the transcription factor NFAT in Jurkat cells treated with either TPA or ionomycin [95]. Together with the constitutive expression of a functional IL-2R caused by Tax1 in HTLV-1 infected T-cells, these results hypothesized that the aberrant activation of an IL-2/IL-2R autocrine loop contributes to the proliferation of infected and leukemic T-cells *in vivo*. However, subsequent studies revealed that most of HTLV-1-infected and leukemic cell lines derived from ATL patients except for HUT102, do not express significant levels of IL-2 [96,97]. Thus, the roles of the IL-2/IL-2R autocrine loop in HTLV-1 mediated T-cell transformation *in vitro* and the leukemogenesis *in vivo *are unclear. Unlike HTLV-1, HTLV-2-infected T-cell lines constitutively produce IL-2 in the culture supernatant, and Tax2 without any additional stimulation activates IL-2 gene expression through NFAT in Jurkat cells [98]. Moreover, cyclosporine A (an inhibitor of NFAT) as well as anti-IL-2R antibodies inhibit the proliferation of HTLV-2-infected T-cell lines [98]. These results suggest that the IL-2/IL-2R autocrine loop is essential for proliferation of HTLV-2-infected cells, and such Tax2-specific activity is a crucial factor for establishing persistent HTLV-2 infection *in vivo*.

It is likely that other factors induced by Tax1 would be beneficial for the survival and proliferation of HTLV-1 infected T-cells *in vivo *through regulating T-cell functions such as cell survival, cell motility, adhesion, and tissue distribution. For instance, OX40 and 4-1BB, by inducing a cell to cell interaction, could further augment NF-κB activity, making a positive feedback loop, which would be important for the maintenance of NF-κB activity in HTLV-1-infected cells that express a low level of Tax1 *in vivo *[99].

### PDZ domain containing proteins

PDZ (PSD-95/Discs Large/ZO-1) domain containing proteins bind to the PDZ domain binding motif (PBM) which is typically present at the carboxyl-terminus of target proteins [100]. One major structural difference between Tax1 and Tax2 is the presence of a PBM at the C-terminus of Tax1, but not Tax2 (Figure 2). It has been shown that deletion of the PBM from HTLV-1 (HTLV-1/ΔPBM) abrogates the persistent HTLV-1 infection in rabbits, whereas the PBM is dispensable for IL-2-dependent immortalization of primary human T-cells [101]. How does the Tax1 PBM play a role in persistent HTLV-1 infection *in vivo *without affecting the IL-2-dependent immortalization of primary T-cells? Interestingly, the deletion of PBM prominently reduces IL-2-independent growth of CTLL-2 cells induced by Tax1 [57]. Taken into account that the steady state level of IL-2 *in vivo *is generally too low to support IL-2-induced T-cell proliferation, the reduced requirement of IL-2 induced by Tax1 through PBM may explain the selectively defective function of HTLV-1/ΔPBM *in vivo*.

It is unclear how the Tax1 PBM contributes to inducing IL-2-independent growth of T-cells. The addition of Tax1 PBM to the C-terminus of Tax2 in the context of HTLV-2 significantly increases the proliferation of primary human T-cells infected with the virus *in vitro *[101]. Thus, Tax1 PBM may engage in the cell growth promoting activity of Tax1. In addition, it should be noted that the Tax1 PBM has an activity to induce micronuclei in Tax1 expressing cells [101-103]. Thus, the Tax1 PBM may have an activity associated with the genomic instability observed in HTLV-1 infected cells.

Similar to the pathogenesis differences between HTLV-1 versus HTLV-2, limited subtypes of human papilloma viruses (HPVs) such as HPV16 and 18 are associated with cervical cancers. Interestingly, the E6 oncoproteins from only high risk HPVs, but not low-risk HPVs have a PBM [100]. A similar pattern of subtype specific oncogenesis is also observed for human adenovirus type 9. Intriguingly, the PBMs from the oncogneic HPV E6 and the adenovirus E4-ORF1 can efficiently substitute for the Tax1 PBM in transforming CTLL-2 cells (submitted for publication). Thus, the targeting of PDZ domain containing proteins is likely to contribute an important mechanism to cellular transformation and pathogenesis by tumorigenic viruses.

Tax1 has been reported to interact with several PDZ domain containing proteins including Dlg1, Scribble, MAGI-3, TIP-1, IL-16 precursor protein, and Erbin [59,104-111]. Below, we discuss the possible involvement of Dlg1 and Scribble in Tax1 function. These proteins are believed to play important roles in the regulation of cell polarity, proliferation, and apoptosis.

### Dlg1

Dlg1 is a mammalian homologue of Drosophila discs-large (dlg) and a member of the membrane-associated guanylate kinase (MAGUK) family proteins [112]. Homozygous *dlg *mutations in Drosophila cause neoplastic overgrowth of imaginal disc epithelia and embryonic lethality, establishing *dlg *as a tumor suppressor gene in Drosophila. In mammalian epithelial cells, Dlg1 localizes at adherence junctions (AJ) and is involved in the establishment of AJ as well as tight junctions. Following T cell receptor (TCR) activation in T cells, Dlg1 is transiently translocated to the immune synapses where it functions as a scaffold coordinating the activities of signaling proteins such as Lck, Zap70, Vav, WASP, and p38 kinase [113-116]. T cells from Dlg1 knockout mice show a hyperproliferative response to TCR stimulation, although proximal TCR signaling events such as tyrosine phosphorylaion of signaling molecules, calcium mobilization, and IL-2 production are indistinguishable from wild type T cells, indicating that Dlg1 functions as a negative regulator of T cell proliferation [117].

Consistent with its tumor suppressive activity, the overexpression of Dlg1 in NIH-3T3 cells induces cell cycle arrest in G1, and this arrest can be overcome by Tax1 in a PBM dependent manner, indicating that Tax1, through direct binding, interferes with the growth-suppressive activity of Dlg1 [106]. Although it has not been elucidated yet how Tax1 inactivates Dlg1 function, Tax1 induces the hyperphosphorylation of Dlg1 by an unknown mechanism, and alters its subcellular localization from the detergent soluble to the detergent insoluble fraction [59]. Unlike Tax1, E6 inactivates Dlg1 by ubiquitination mediated proteosomal degradation [118]. Consistent with the fact that Dlg1 deficiency in T cells augments cell proliferation, Dlg1 knockdown by short hairpin RNA in CTLL-2 cells augments Tax1 mediated transformation, although the knockdown alone cannot rescue the transforming activity of a Tax1 PBM mutant incapable of binding to Dlg1 [119]. These results suggest that the inactivation of Dlg1 is required for Tax1 mediated transformation of CTLL-2, but this is not sufficient; and other PDZ domain containing proteins are involved in the activity of PBM.

### Scribble

Scribble is a member of the LAP (leucine rich and PDZ domain) family of proteins and functions as a cell polarity protein in cooperation with Dlg1 [112]. Like *dlg*, loss-of-function mutation of *scrib *in Drosophila results in aberrant proliferation and abnormal cell polarity/architecture of epithelial cells, indicating a role for Scribble as a tumor suppressor. In mammalian epithelial cells, Scribble and Dlg1 also form a scaffolding complex, regulating apical-basal polarity. In T cells, the Scribble complex regulates T cell polarity and morphology during migration and immunological synapse formation [115].

Tax1 binds to Scribble in a PBM dependent manner, although one report suggests there are both PBM-dependent and -independent modes of Tax1-Scribble binding [107,108]. Scribble is diffusely localized at the plasma membrane of HTLV-1-uninfected T-cell lines, whereas it colocalizes with Tax1 as small or large aggregates at the plasma membranes, suggesting that Tax1 induces aberrant clustering of Scribble, thereby altering its functions in HTLV-1-infected cells [107,108]. Although Scribble is targeted for ubiquitin mediated degradation by high-risk HPV E6, there seems to be no obvious degradation of Scribble in HTLV-1 transformed cells, suggesting that alteration of the subcellular localization of Scribble by Tax1 is the main mechanism to inactivate its function. Scribble over expression in Jurkat cells suppresses TCR-induced NFAT activity, and this suppression is relieved by Tax1 in a PBM dependent manner, indicating that Tax1 interferes with Scribble function although the significance of this finding in infected T-cells remains unclear [107].

While it is very likely that viral oncoproteins including Tax1 inactivate the tumor suppressive functions of PDZ domain proteins, it remains possible that they might positively utilize such PDZ proteins to transform cells. This idea was raised in order to explain the function of E4-ORF1, since the loss of Dlg1 apparently reduces the transforming activity of E4-ORF1 [120]. By analogy, Tax1 might take advantage of PDZ domain containing protein(s) to localize at certain cellular organelles, such as cell membranes, in order to activate signaling pathways important for cell growth and survival. Thus, the understanding of the whole picture of Tax1 PBM function in HTLV-1 mediated T-cell transformation is still incomplete, and further studies are needed.

### Activation of PI3K and Akt pathway

PI3K and its downstream kinase Akt are activated in T cells by many cytokines including IL-2; this pathway provides cell survival and growth signals [121]. Activated PI3K phosphorylates phosphatidylinositol 4,5-bisphosphate to produce phosphatidylinositol 3,4,5-trisphosphate (PIP3) which binds and activates Akt. Activated Akt in turn phosohorylates its downstream substrates which are involved in cell survival and cell growth. On the other hand, PIP3 phosphatases, such as phosphatase and tensin homolog deleted on chromosome 10 (PTEN) and Src homology 2 domain containing inositol polyphosphate phosphatase-1 (SHIP-1), dephosphorylate PIP3 to downregulate Akt activity.

In many cancers, the PI3K/Akt pathway is aberrantly activated by several means, including the gain of function mutation in PI3K and Akt, the loss of PTEN, and the constitutive activation of upstream signaling molecules such as the mutation of *ras *[122]. In both HTLV-1 transformed and ATL cells, the PI3K/Akt pathway is constitutively active [123,124]. LY294002 (an inhibitor of PI3K) or AKT inhibitor II induces cell cycle arrest at G1 phase in HTLV-1 transformed cells through p27/kip1 accumulation, and they subsequently induce caspase-9 dependent apoptosis [101]. These findings indicate that PI3K/Akt activation by Tax1 is critically involved in the growth of HTLV-1-infected T-cells [125].

Several distinct mechanisms for Tax1 to activate PI3K/Akt have been reported. Tax1 frees a catalytic p110α subunit of PI3K complex from an inhibitory subunit p85α through direct binding to p85α [124]. Tax1 also down-regulates the expression of PTEN and SHIP-1 through RelA-mediated sequestration of the transcriptional coactivator p300 from the promoters of PTEN and SHIP-1 [126]. In addition, Tax1 through the CREB/ATF-1 pathway activates Akt in 293T cells, although in this setting the precise mechanism remains unclear [127].

The mammalian target of rapamycin (mTOR) is one of the crucial downstream targets of Akt which is used to promote cell survival and growth mainly through the stimulation of translational initiation [128]. Rapamycin, an inhibitor of mTOR kinase activity, inhibits the phosphorylation of p70S6 kinase and 4E-BP1, thereby inducing growth inhibition and G1 cell cycle arrest of HTLV-1 transformed cells. These findings are consistent with mTOR activation being important for Tax-induced cell cycle progression [129]. In addition to mTOR, AP-1, NF-κB, β-catenin, and HIF-1 are activated by Tax1 through PI3K/Akt in HTLV-1-infected T-cells, and these factors also seem to be involved in HTLV-1 mediated T-cell transformation [124,125,127,130].

Paradoxical to the virus' transforming activity, Tax1 expression in or HTLV-1 infection of human cells (HeLa, SupT1 T-cell line) has been observed in some settings to induce cell cycle arrest at the G1 phase through the induction of p27/kip1 and p21/waf1 [131]. This is often associated with the premature activation of the anaphase-promoting complex (APC) [132]. This type of growth inhibition by Tax1 or HTLV-1 infection is abrogated by elevated Akt activity [133]. These results suggest that Akt activation by Tax1 in cells may not be sufficient to inactivate p27/kip1 and p21/waf1 functions, and that additional inactivation of p27/kip1 and p21/waf1 by genetic and/or epigenetic alterations could be essential for HTLV-1 to transform T-cells. It should be noted that 8 to 9 weeks after infection with HTLV-1, primary human T-cells can start to proliferate [101]; this lag time may be due to the interval of time needed to obtain genetic and/or epigenetic changes in order to escape cell cycle arrest induced by Tax1.

### Tax3 and Tax4

Like HTLV-1 and HTLV-2, HTLV-3 and HTLV-4 encode Tax3 and Tax4, respectively. In their cognate viruses, Tax3 and Tax4 could play major roles in T-cell immortalization and persistent infection (Figure 2) [15,16]. Amino acid comparisons show that Tax3, but not Tax4, has a PBM at its C-terminus, and can bind to a PDZ domain derived from Dlg4 [15]. In addition, Tax3 and Tax4 show more homology to Tax2 than Tax1 in the LZR region which is important for the noncanonical activation of NF-κB. However, it has not been verified whether Tax3 and/or Tax4 activate the noncanonical NF-κB pathway. Therefore, the PBM and the LZR classify these four HTLVs into at least three distinct groups. Taken together, the PBM and LZR motifs could play significant roles in the respective life cycles of the HTLVs and contribute to their pathogenesis.

### HBZ

Although the *tax *gene plays central roles in the immortalization and persistence of virus infected cells, its expression is inactivated in approximately 60% of *in viv*o ATL cases by mutation of the coding region and/or the transcriptional silencing through epigenetic mechanisms such as DNA methylation of the 5' LTR [134-139]. These findings suggest that Tax1 is not needed in the maintenance of the leukemic stage in some ATL cases. The frequent inactivation of the *tax *gene was originally interpreted to imply the dispensability of any HTLV genes for the maintenance of the leukemic stage in certain ATL cases. This was a reasonable interpretation since the expression of viral genes other than *tax *was usually not detected in ATL cells. Recent studies, however, showed that the HTLV-1 basic leucine zipper factor (HBZ) encoded by the virus in an antisense orientation may play a critical role in the malignant proliferation of ATL cells (Figure 1) [140]. The expression of HBZ gene is detected in all ATL cases, and this is due to the usage of the promoter in the 3' LTR of HTLV-1 gene which is not inactivated in the ATL cells [141,142]. Short hairpin RNA mediated knockdowns of HBZ expression in both ATL and HTLV-1 transformed cell lines reduce their proliferation [141,143]. Moreover, transgenic mice expressing HBZ under the control of the CD4 promoter/enhancer display increased numbers of CD4-positive T-cells in the spleen, and augmented proliferation of thymocytes after anti-CD3 stimulation [141]. Thus, these findings indicate that HBZ has a growth promoting activity, and could be involved in the malignant proliferation of ATL cells *in vivo*, although the precise molecular mechanism for these findings is still unclear. HTLV-2 also encodes a HBZ like protein, designated as the antisense protein of HTLV-2 (APH-2) [144]. Interestingly, unlike HBZ, APH-2 does not have a leucine zipper motif which is essential for various HBZ functions. Thus, it is important to study whether the HTLV-2 APH-2 protein has a growth promoting activity in T-cells like HBZ in order to understand better how these two viruses show distinct pathogenicities.

## Conclusion

**Figure 3:**
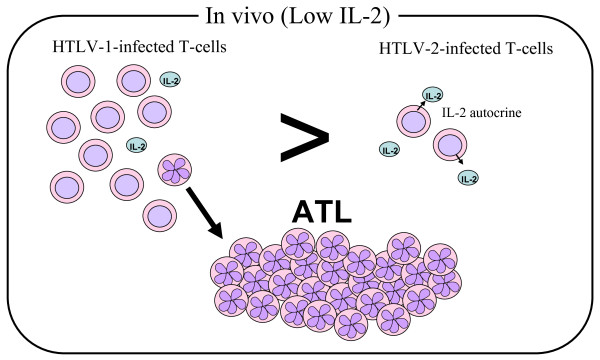
**A model for HTLV-1-specific pathogenesis**. The amounts of IL-2 or similar T-cell growth-promoting cytokines are low *in vivo*. In such environment, HTLV-1 infected cells proliferate more efficiently than HTLV-2 infected cells, and have greater probability to acquire genetic and/or epigenetic mutations. In addition, increased proliferation of HTLV-1 infected T-cells would effectively deteriorate the host immune system. Once such mutated cells accumulate with a reduced host immune activity, HTLV-1-infected T-cells can grow monoclonally, resulting in ATL development. For HAM/TSP, an increase in HTLV-1-infected cells *in vivo *induces more immune response to HTLV-1, especially to Tax1, resulting in HAM/TSP development through an autoimmune mechanism.

This review article summarizes our current view pertaining to the molecular mechanism(s) of HTLV-1 mediated T-cell transformation and persistent infection. In our opinion, these mechanism(s) shed light on viral pathogenesis, and offer insights into differences in HTLV-1 Tax1 and HTLV-2 Tax2 function. Based on current information, we propose the following simplified model (Figure 3) which does not incorporate other potentially important factors such as oncogenic microRNAs [145-147]. HTLV-1-infected T-cells grow equivalently to HTLV-2-infected cells in environments with sufficient amount of IL-2 or other T-cell growth promoting cytokines *in vivo*, but HTLV-1 infected cells under conditions of low cytokines can grow much more efficiently than HTLV-2-infected cells. Such growth advantage of HTLV-1 infection would cause more expansion of infected cells *in vivo*, resulting in increased probability of acquiring genetic alterations, followed by clonal expansion of altered cells, and eventually leading to ATL development. It should be noted that high HTLV-1 proviral load (high numbers of infected cells) is a tightly-linked risk factor for the development of ATL. Such cytokine-independent (or less-dependent) growth properties of HTLV-1 infected T-cells are mediated by Tax1, possibly cooperatively with HBZ. To induce IL-2-independent growth of T-cells, Tax1 has two activities distinct from Tax2: the activation of the noncanonical NF-κB2 pathway and as-yet-uncharacterized signals through PDZ domain-containing proteins. These two activities are already known to play crucial roles in hematopoietic malignancies including leukemia and lymphoma and carcinogenesis induced by high-risk HPVs. This model would also be applicable to the pathogenesis of HAM/TSP, since high proviral loads are also tightly-linked risk factors for HAM/TSP. In HAM/TSP, the increased expansion of HTLV-1 infected T-cells would further raise high immune response to the virus, especially to Tax1, resulting in the development of diseases possibly through already proposed autoimmune mechanism(s). Collectively, we believe that further comparisons of Tax functions from the four human HTLVs will promote greater understanding of viral pathogenesis. In addition, therapies targeted against functions specific to Tax1 could be promising for the treatment of HAM/TSP and certain ATL patients.

## Competing interests

The authors declare that they have no competing interests.

## Authors' contributions

MH and MF cooperatively wrote and edited this review. Both authors read and approved the final manuscript.
